# Serum Pro‐N‐Cadherin Is a Marker of Subclinical Heart Failure in the General Population

**DOI:** 10.1161/JAHA.122.028234

**Published:** 2023-03-09

**Authors:** Paul Durham Ferrell, Kristianne Michelle Oristian, Ishaan Puranam, Salvatore Vincent Pizzo

**Affiliations:** ^1^ Department of Pathology Duke University School of Medicine Durham NC; ^2^ Department of Radiation Oncology Duke University School of Medicine Durham NC; ^3^ Department of Biomedical Engineering Duke University Pratt School of Engineering Durham NC

**Keywords:** biomarkers, BNP, cardiovascular disease, heart failure, NT‐proBNP, pro‐N‐cadherin, Biomarkers, Fibrosis, Heart Failure, Remodeling

## Abstract

**Background:**

We recently reported aberrant processing and localization of the precursor PNC (pro‐N‐cadherin) protein in failing heart tissues and detected elevated PNC products in the plasma of patients with heart failure. We hypothesize that PNC mislocalization and subsequent circulation is an early event in the pathogenesis of heart failure, and therefore circulating PNC is an early biomarker of heart failure.

**Methods and Results:**

In collaboration with the Duke University Clinical and Translational Science Institute's MURDOCK (Measurement to Understand Reclassification of Disease of Cabarrus and Kannapolis) study, we queried enrolled individuals and sampled 2 matched cohorts: a cohort of individuals with no known heart failure at the time of serum collection and no heart failure development in the following 13 years (n=289, cohort A) and a matching cohort of enrolled individuals who had no known heart failure at the time of serum collection but subsequently developed heart failure within the following 13 years (n=307, cohort B). Serum PNC and NT‐proBNP (N‐terminal pro B‐type natriuretic peptide) concentrations in each population were quantified by ELISA. We detected no significant difference in NT‐proBNP rule‐in or rule‐out statistics between the 2 cohorts at baseline. In participants who developed heart failure, serum PNC is significantly elevated relative to those who did not report development of heart failure (*P*<0.0001). Receiver operating characteristic analyses of PNC demonstrate diagnostic value for subclinical heart failure. Additionally, PNC has diagnostic potential when comparing participants with no reported heart failure risk factors from cohort A to at‐risk participants from cohort B over the 13‐year follow‐up. Participants whose PNC levels measure >6 ng/mL have a 41% increased risk of all‐cause mortality independent of age, body mass index, sex, NT‐proBNP, blood pressure, previous heart attack, and coronary artery disease (*P*=0.044, n=596).

**Conclusions:**

These data suggest that PNC is an early marker of heart failure and has the potential to identify patients who would benefit from early therapeutic intervention.

**Nonstandard Abbreviations and Acronyms**
MURDOCK: Measurement to Understand Reclassification of Disease of Cabarrus and Kannapolis
PNC: precursor pro‐N‐cadherin

**Clinical Perspective**

**What Is New?**

- Serum pro‐N‐cadherin products are significantly elevated in patients with subclinical heart failure and add diagnostic/prognostic value to traditional heart failure risk factors.

**What Are the Clinical Implications?**

- While more studies are necessary to establish optimal threshold values for serum pro‐N‐cadherin as a diagnostic/prognostic indicator in subclinical heart failure, elevated pro‐N‐cadherin has the potential to become a useful tool to stratify and evaluate patients for risk of disease.

Heart failure is the leading cause of morbidity and mortality in the developed world and accounts for 1 in 8 deaths in the United States according to the Centers for Disease Control and Prevention.^1^ Despite this, the major molecular mechanisms of heart failure remain elusive, and treatment for heart failure is almost exclusively designed to alleviate symptoms after their onset. Even the diagnosis of heart failure suffers from a lack of consensus symptoms and biomarkers that define the onset of disease.^2^ This lack of understanding behind the molecular pathogenesis of heart failure has translated to a corresponding lack of molecular biomarkers that reflect early cardiac remodeling and accurately predict the development of heart failure before the onset of symptoms.

Current definitions of heart failure staging predict that ≈50% of the general population aged >45 years fall within stage A and B heart failure.^3^ The standard of care serological biomarker for ruling in or ruling out heart failure is BNP (B‐type natriuretic peptide) or its precursor, NT‐proBNP (N‐terminal pro‐B‐type natriuretic peptide). NT‐pro/BNP (NT‐proBNP and BNP, inclusive) functions as a natriuretic peptide that compensates for cardiac wall stress by inducing vasodilation, leading to a reduction in cardiac filling pressure and increased cardiac output.^4^ This suggests that serum BNP levels are a surrogate for measuring cardiac wall stress and increase after biochemical compensation pathways are triggered. Clinically, NT‐pro/BNP perform better for ruling out than ruling in heart failure with similar predictive value.^1^, ^5^, ^6^ In part, this is because of a lack of consensus rule‐in and rule‐out standards. However, the analysis of serum NT‐pro/BNP must also be considered in the context of many other comorbidities and influential variables. These include age, sex, race, obesity, and other cardiovascular and noncardiovascular diseases and syndromes that can raise or lower NT‐pro/BNP in the blood.^7^, ^8^, ^9^, ^10^, ^11^, ^12^, ^13^ Unfortunately, the earliest stage of heart failure in which NT‐pro/BNP values may be elevated is stage B.^1^, ^14^, ^15^ In a study evaluating the prognostic value of NT‐proBNP for death and cardiovascular events in both healthy subjects and subjects with stage A/B heart failure, the authors found that NT‐proBNP was not predictive of morbidity or mortality in healthy subjects.^16^ They further found that when comparing study participants with NT‐proBNP above the 80th percentile, the difference between those with stage A/B heart failure and healthy controls only amounted to 11.9% (24.7% versus 12.8%, respectively).^16^ This “gray zone” of overlap between healthy individuals and those with stage A/B heart failure highlights a clear unmet need for an accurate and specific biomarker that can be used to screen and detect at‐risk individuals.

In previous work, we found that defective processing of N‐cadherin in heart failure leads to cell‐surface expression and aberrant localization of the precursor form of N‐cadherin (PNC [pro‐N‐cadherin]) on myofibroblasts and at intercalated discs in failing heart tissue.^17^ With the prodomain intact, homophilic interactions found between N‐cadherin in normal cellular junctions become sterically hindered, putatively disallowing the normal coordinated contractile functions of the cardiac muscle.^18^ Consistent with our findings, Chen et al^19^ recently reported a novel variant of N‐cadherin identified in a 12‐year‐old girl in whom a point mutation resulted in retained prodomain at the cell surface. The mutant N‐cadherin had significantly impaired adhesion efficiency, and despite heterozygous expression of the mutation, the patient developed dilated cardiomyopathy and died of her disease at age 13 years.^19^ We further showed that the prodomain peptide can be detected in the serum of patients with heart failure.^17^ Here, we evaluate the expression of soluble pro‐N‐cadherin as a biomarker for subclinical heart failure as compared with the standard marker, NT‐proBNP.

## Methods

The authors declare that all supporting data are available within the article and its supplemental files.

### Study Participants

The study population included 690 participants within the MURDOCK (Measurement to Understand Reclassification of Disease of Cabarrus and Kannapolis) study community registry and biorepository.^20^ Collection of serum as part of the MURDOCK study has been described.^20^, ^21^ All participants in the study population reported no heart failure at baseline, as indicated by response to the MURDOCK study enrollment questionnaire. More information about the MURDOCK study storefront is included in Data S1. A control cohort with no reported heart failure at baseline and no reported heart failure over a 13‐year follow‐up was identified (cohort A, n=289) from within the study population. A second cohort with no reported heart failure at baseline but later reported heart failure at any time during a 13‐year follow‐up was identified (cohort B, n=307) from within the study population. A “low‐risk” subgroup of cohort A was identified by excluding participants with coronary artery disease, high blood pressure, previous heart attack, atrial fibrillation, or NT‐proBNP levels above the age‐dependent rule‐in cutoffs for heart failure at enrollment. The age‐dependent rule‐in consensus values of 450, 900, and 1800 pg/mL for ages <50, 50 to 75, and >75 years, respectively, were used in this study. A “high‐risk” subgroup of cohort B was identified that included participants who reported at least 1, 2, or 3 heart failure risk factors. Risk factors were defined as coronary artery disease, high blood pressure, or previous heart attack reported at enrollment. Where indicated, low‐risk subgroup A was compared with high‐risk subgroup B. Insufficient samples prevented measurement of NT‐proBNP in 6 samples from cohort B that were excluded from analysis.

The MURDOCK Community Registry and Biorepository and related ancillary studies are approved by the institutional review boards of both Duke University Medical Center (Durham, NC) and Carolinas HealthCare System (Charlotte, NC). All patients provided written informed consent for the collection of biological samples and use of their clinical data. The current analyses were approved by the Duke University Medical Center Institutional Review Board.

### Enzyme‐Linked Immunosorbent Assays

Detection and quantification of serum pro‐N‐cadherin by ELISA has been described.^17^ Detection and quantification of serum NT‐proBNP was performed according to the manufacturer's recommendations (R&D Systems, Minneapolis, MN; DY3604‐05).

### Statistical Analysis

Prism version 9.4.0.673 (GraphPad Software, San Diego, CA) was employed for statistical analysis. Welch's *t*‐test was used to evaluate the significance of serum values between the 2 cohorts. Receiver operating characteristic (ROC) curves were calculated by the Wilson/Brown method with 95% CIs. Relationships between PNC, age, body mass index (BMI), and NT‐proBNP were evaluated by simple linear regression. Welch's *t*‐test was performed to evaluate differences of PNC values between male and female sexes. Distribution of age, BMI, weight, and blood pressure were evaluated using the Mann–Whitney test except diastolic blood pressure, which was evaluated using Welch's test following a test of normality. Proportion of racial and ethnic groups was evaluated using Fisher's exact test. Survival curves with hazard ratios (HRs) were generated using the log‐rank test. Adjusted HRs for all‐cause mortality and development of heart failure were generated using the Cox proportional hazards ratio (*P*<0.05*, *P*<0.01**, *P*<0.001***, *P*<0.0001****).

## Results

### Study Population and Definition of Cohorts

The study population comprised 690 participants from the MURDOCK study at the Duke Clinical and Translational Science Institute (Data S1).^20^ All participants in the study population enrolled at time=0 as members of the general population with no known heart failure. They were subsequently followed for 1 to 13 years by a self‐report style questionnaire in which any health conditions that developed were noted. Cohort A (n=345) is defined by those who did not report the development of heart failure at any time following enrollment. Cohort B (n=345) is defined by those who indicated no known heart failure at time of enrollment and serum collection (time=0) but reported development of heart failure on a subsequent follow‐up. NT‐proBNP levels were used to corroborate the heart failure status of each cohort. Cohorts were populated by the Duke Clinical and Translational Research Institute and reviewed for exclusion criteria by study investigators. Cohorts were further refined by the following exclusions: participants with no follow‐ups and participants who reported “yes,” “I don't know,” or “null” to heart failure at baseline. Cohorts A and B hereafter will refer to cohorts after exclusion refinement on the basis of the above criteria (Table 1). Population dynamics of cohorts A and B after exclusion refinement do not differ significantly from those of the unrefined cohorts.

**Table 1:** Demographic Summary of Participants Curated for Study

	All Participants	Cohort A	Cohort B	*P* value	NC,* %
N	690	345	345		
N (after exclusion refinement)	596	289	307		
Age, y	68 [22–95]	68 [27–95]	68 [22–95]	0.7615	
BMI	30 [16–82]	28 [17–60]	29 [16–82]	0.0004§	
Weight, lbs	188 [92–506]	179 [92–404]	197 [95–506]	0.0010§	
Systolic blood pressure, mm Hg	133 [72–231]	132 [81–231]	134 [72–197]	0.3162	
Diastolic blood pressure, mm Hg	75 [44–126]	76 [52–126]	74 [44–120]	0.0815	
Male‐identified individuals	298 (43)	149 (43)	149 (43)	>0.9999	49
Female‐identified individuals	392 (57)	196 (57)	196 (57)	>0.9999	51
Hispanic	15 (2)	9 (3)	6 (2)	0.6032	12
Black	116 (17)	58 (17)	58 (17)	>0.9999	21
AAPI	2 (0)	0 (0)	2 (0.5)	0.4993	2
Native American	4 (0.5)	2 (0.5)	2 (0.5)	>0.999	0
Non‐Hispanic White	544 (79)	275 (80)	269 (78)	0.6413	61
Majority sample collection year†	2011 [2009–2016]	2009 [2009–2016]	2011 [2009–2016]	…	
Follow‐up period‡, y	7.6±3 [1–13]	7.4±3.5 [1–13]	7.8±3.2 [1–13]	…	
Cardiovascular risk factors	0.9	0.6	1.1	<0.0001§	

Unless otherwise indicated, values are reported as: arithmetic mean [range]. Units, where applicable, are indicated in parentheses. Racial and ethnic identity and biological sex are reported as n (%). AAPI indicates Asian American and Pacific Islander; and BMI, body mass index.

* For reference, US Census Bureau statistics for the 2010 Census in Kannapolis, NC, are provided.

† Majority sample collection year is reported as arithmetic mode of the calendar years in which samples were collected for participants in this study [range].

^‡^ Follow‐up period is reported as arithmetic mean±SD [range]. Cardiovascular risk factors are reported as arithmetic means, given that each participant may have any combination of each of 3 predetermined cardiovascular risk factors at time of sample collection: high blood pressure, prior heart attack, and coronary artery disease and presence of a given risk factor is weighted with a value of 1. *P* values are provided comparing the distribution (age, BMI, weight, blood pressure) or proportion (race, ethnicity) where appropriate between cohort A and cohort B using Mann–Whitney, Welch's, or Fisher's exact test.

^§^ Significant differences (*P*<0.05).

### Population Dynamics of Cohorts A and B

**Figure 1:**
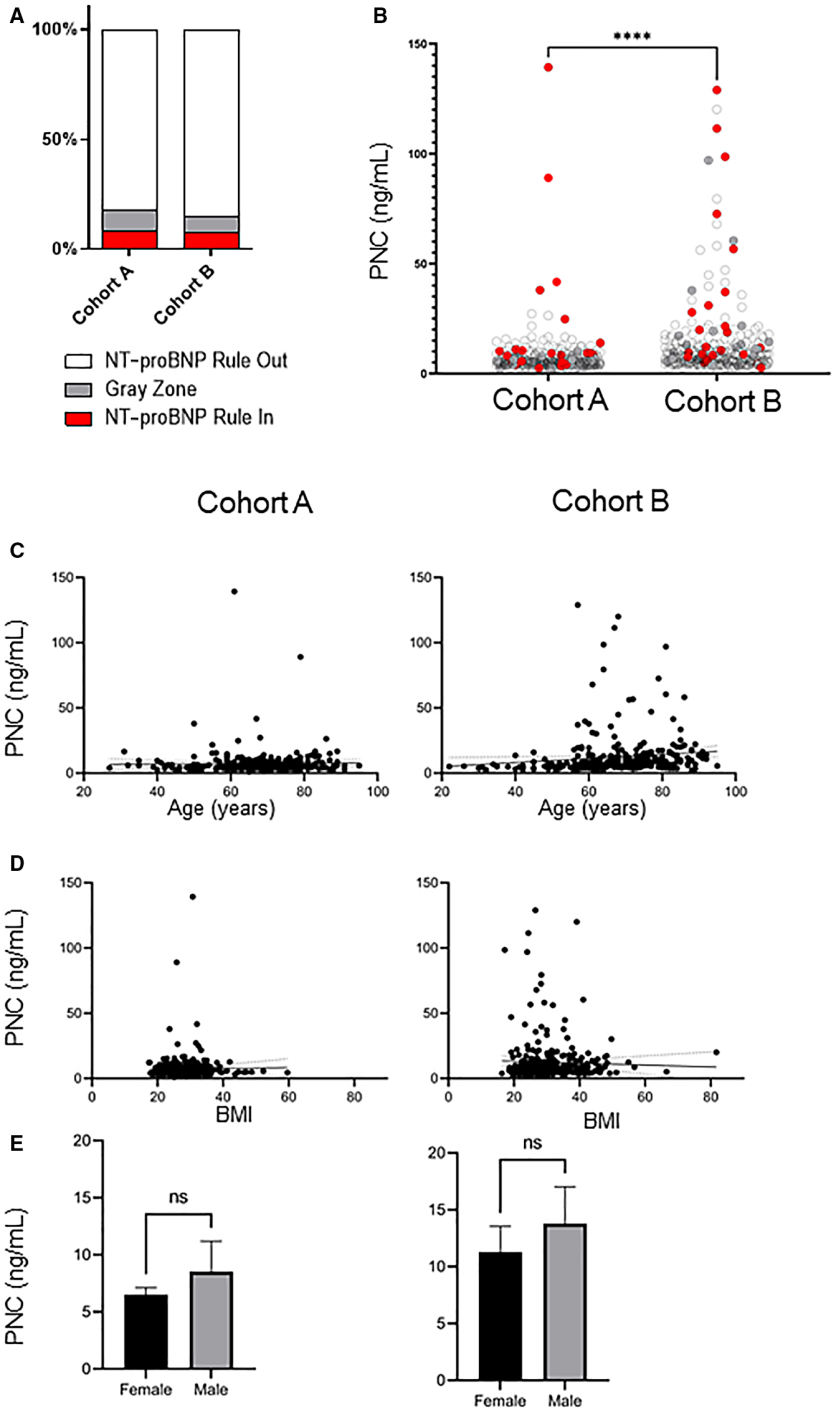
Relationship of PNC to NT‐proBNP and potential confounding variables. **A**, Graphical representation of the percent distribution of NT‐proBNP rule‐in, rule‐out, and “gray zone.” **B**, PNC values were analyzed between cohort A and cohort B by unpaired *t*‐test with Welch's corrections. Cohort B has significantly higher PNC levels with a mean value of 12.38 ng/mL relative to cohort A mean value of 7.37 ng/mL (n=596; *P*<0.0001). Red dots represent participants who meet the NT‐proBNP heart failure rule‐in criteria, white/transparent dots fall within the NT‐proBNP rule‐out criteria, and gray dots represent participants whose NT‐proBNP falls within the gray zone. **C**, Simple linear regression analysis of age versus PNC values for cohort A and cohort B. No correlation to age and PNC levels is observed in cohort A (n=289; slope, 0.0132; *r* ^2^=0.0002; slope, non‐0; *P*=0.79), but a slight correlation is found in cohort B (n=307; slope, 0.1551; *r* ^2^=0.0148; slope, non‐0; *P*=0.03). **D**, Simple linear regression analysis of BMI versus PNC values. The slope of either cohort A (n=289; slope, 0.0298; *r* ^2^=0.0003; slope, non‐0; *P*=0.78) nor cohort B (n=307; slope, −0.0722; *r* ^2^=0.0013; slope, non‐0; *P*=0.54) deviated significantly from 0 for BMI versus PNC values. **E**, Correlation between PNC values and sex was analyzed using an unpaired *t*‐test with Welch's correction. Neither cohort A (n=289; mean female, 6.50 ng/mL; mean male, 8.53 ng/mL; *P*=0.14) nor cohort B (n=307; mean female, 11.29 ng/mL; mean male, 13.79 ng/mL; *P*=0.21) differed in PNC values between women versus men. BMI indicates body mass index; NT‐proBNP, N‐terminal pro‐B‐type natriuretic peptide; and PNC, precursor pro‐N‐cadherin.

The median ages of cohorts A and B are 68 (27–95) and 68 (22–95) years, respectively. The ratio of men to women in each cohort was matched, 57% women and 43% men. Cohort B had higher reporting of high blood pressure: 50.7% of cohort A and 68.5% of cohort B reported high blood pressure at enrollment. However, there was no significant difference in measured mean systolic (cohort A, 132.0 versus cohort B, 134.0; *P*=0.2333) or diastolic (cohort A, 76 versus cohort B, 74; *P*=0.0815) blood pressure at the time of enrollment between the 2 cohorts. Cohort B reports overall higher cardiovascular risk factors. The mean BMI was slightly higher in cohort B at 29 (16–82) relative to cohort A at 28 (17–60). Both groups were well within the margin of error for parameters of NT‐proBNP levels expected from the general population with no diagnosis of heart failure. We selected a study‐designated rule‐out cutoff of 300 pg/mL, consistent with a review of the NT‐proBNP literature and within the quantifiable limits of the assay used for this study.^22^ Of cohort A, 82.4% were below the study‐designated rule‐out cutoff <300 pg/mL NT‐proBNP for heart failure and 8.0% were above the age dependent cut offs previously listed. Of cohort B, 85.4% were below the rule‐out cutoff (<300 pg/mL NT‐proBNP) for heart failure, and 7.3% were above the age‐dependent cutoffs. Both cohorts show a similar proportion of expected rule‐in and rule‐out NT‐proBNP values (Figure 1A and 1B; Figure S1).

### Relationship of Soluble PNC to NT‐proBNP and Potential Confounding Variables

A relationship between PNC and age, sex, or BMI, was analyzed. A simple linear regression suggests that neither age nor BMI are correlated with PNC levels within either cohort (Figure 1C and 1D). There is no significant difference in PNC levels between men and women from either cohort A (Figure 1E; *P*=0.1436) or B (Figure 1E; *P*=0.2121).

### Soluble PNC Is a Biomarker of Subclinical Heart Failure

**Figure 2:**
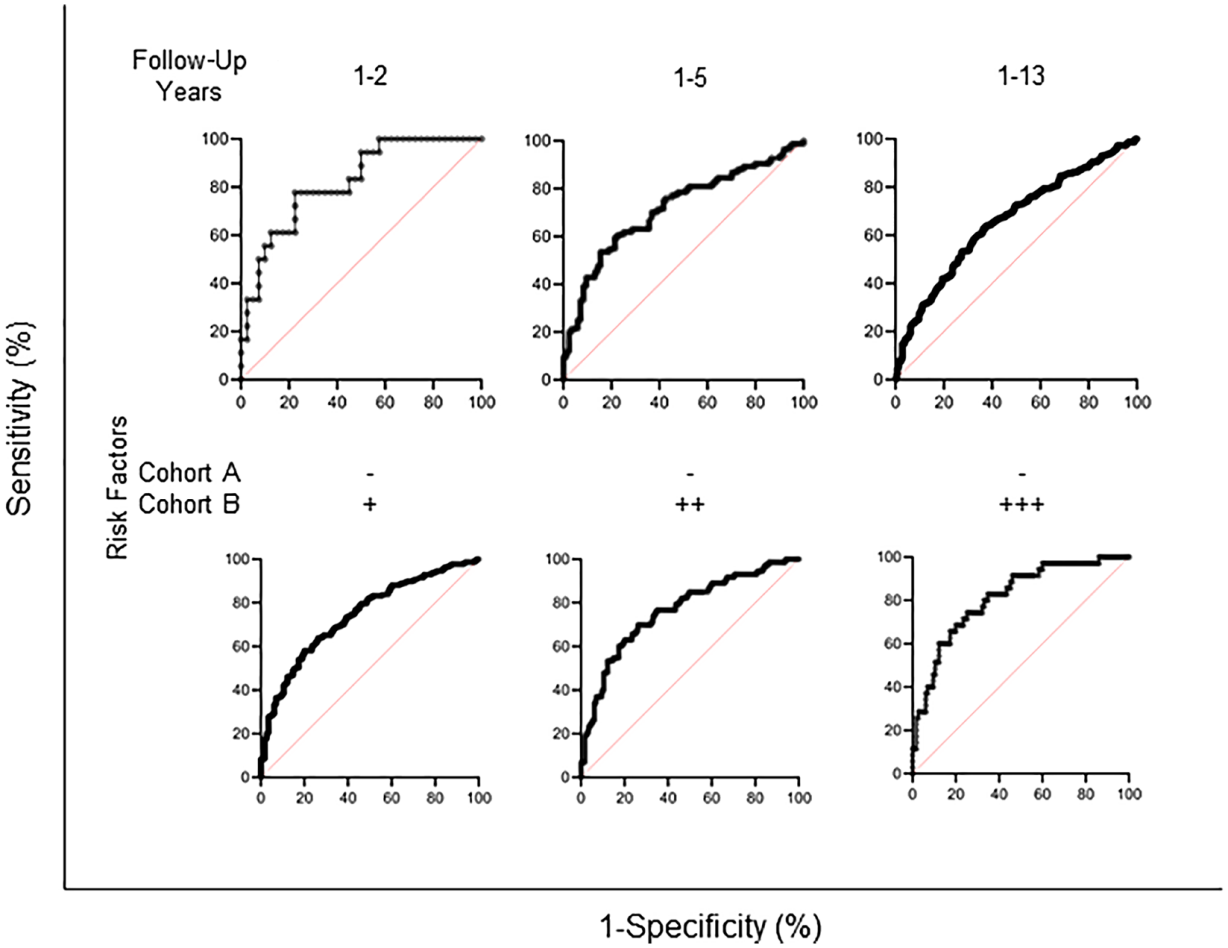
Pro‐N‐cadherin is a biomarker of subclinical heart failure. (Top) ROC analysis using Wilson/Brown method was performed comparing cohort A versus cohort B for participants who followed up within 1 to 2 years, 1 to 5 years, and 1 to 13 years. The AUC is greatest for participants who follow up within 1 to 2 years (total n=58; AUC, 0.82 [95% CI, 0.71–0.93]; *P*=0.0001), followed by 1 to 5 years (total n=168; AUC, 0.72 [95% CI, 0.64–0.79]; *P*<0.0001) and 1 to 13 years (total n=596; AUC, 0.66 [95% CI, 0.62–0.70]; *P*<0.001). (Bottom) ROC analysis using the Wilson/Brown method of participants within cohort A excluding participants who meet the criteria for NT‐proBNP heart failure rule‐in and participants who report coronary artery disease, heart attack, high blood pressure, or atrial fibrillation at the time of blood draw versus cohort B participants who report at least 1, 2, or 3 heart failure risk factors. The AUC is greatest for participants with at least 3 heart failure risk factors (total n=150; AUC, 0.81 [95% CI, 0.73–0.89]; *P*<0.0001), followed by at least 2 risk factors (total n=188; AUC, 0.76 [95% CI, 0.69–0.84]; *P*<0.0001) and at least 1 risk factor (total n=342; AUC, 0.74 [95% CI, 0.69–0.80]; *P*<0.0001). AUC indicates area under the curve; NT‐proBNP, N‐terminal pro‐B‐type natriuretic peptide; and ROC, receiver operating characteristic.

Participants who subsequently reported heart failure have significantly higher levels of soluble PNC in the serum relative to participants who did not subsequently report heart failure following enrollment (Figure 1B). ROC analysis was performed using PNC values to determine diagnostic accuracy for subclinical heart failure. First, we analyzed each cohort with no exclusion criteria applied over follow‐up periods of ≤13 years, ≤5 years, and ≤2 years (Table 2; Figure 2, top). We then performed ROC analysis of all follow‐up times, excluding participants in cohort A who met the heart failure rule‐in criteria for NT‐proBNP levels or reported any heart disease risk factors and compared them with participants who reported at least 1, 2, or 3 study‐designated heart disease risk factors in cohort B (Table 2; Figure 2, bottom). The majority of NT‐proBNP values fall below the quantifiable range of the NT‐proBNP assay; therefore, ROC analysis was not indicated.

**Table 2:** ROC Analyses of Age, BMI, and Cardiovascular Risk Factors Relative to PNC

AUC	1–13 years F/U	1–5 years F/U	1–2 years F/U	Cardiovascular risks ≥1	Cardiovascular risks ≥2	Cardiovascular risks=3
Age, y	0.5072 (0.4608–0.5536)	0.5485 (0.4612–0.6359)	0.5785 (0.4201–0.7368)	0.6440 (0.5904–0.7076)	0.7014 (0.6283–0.7745)	0.6884 (0.6015–0.7754)
BMI	0.5405 (0.5001–0.5809)	0.5837 (0.4965–0.6709)	0.5342 (0.3567–0.7117)	0.6689 (0.6095–0.7283)	0.6638 (0.5835–0.7441)	0.6678 (0.5585–0.7770)
Cardiovascular risks	0.6516 (0.6081–0.6951)	0.6304 (0.5465–0.7142)	0.6146 (0.4483–0.7809)			
PNC	0.6595 (0.6160–0.7029)	0.7158 (0.6375–0.7941)	0.8208 (0.7089–0.9328)	0.7422 (0.6887–0.7957)	0.7649 (0.6942–0.8355)	0.8127 (0.7336–0.8917)
*P* value						
Age, y	0.7614	0.2773	0.3423	<0.0001	<0.0001	0.0008
BMI	0.0466	0.0626	0.6803	<0.0001	0.0002	0.0028
Cardiovascular risks	<0.0001	0.0035	0.1656			
PNC	<0.0001	<0.0001	0.0001	<0.0001	<0.0001	<0.0001
∆AUC						
PNC vs age	0.1523	0.1673	0.2423	0.0982	0.0635	0.1243
PNC vs BMI	0.119	0.1321	0.2866	0.0733	0.1011	0.1449
PNC vs cardiovascular risks	0.0079	0.0854	0.2062			

AUC, *P* value, and change in AUC are reported for each subgroup: participants followed 1–13 years, participants followed 1–5 years, participants followed 1–2 years, and number of cardiovascular risk factors. AUC values are reported as AUC (95% CI). AUC indicates area under the curve; BMI, body mass index; F/U, follow‐up; PNC, precursor pro‐N‐cadherin; and ROC, receiver operating characteristic.

Age, BMI, and number of defined cardiovascular risk factors were also analyzed by ROC curve for each subgroup (Table 2). The area under the curve (AUC), *P* value, and change in AUC relative to PNC was calculated for each subgroup analyzed. When binning for follow‐up times, AUC involving age, BMI, and number of cardiovascular risk factors remained relatively constant; however, the diagnostic ability of PNC with shortened follow‐up times analyzed was enriched. Favorable risk discrimination for developing heart failure using PNC was observed at a follow‐up time of ≤2 years (*P*=0.0001; AUC, 0.82 [95% CI, 0.71–0.93]). Binning cohort B for number of cardiovascular risk factors and comparing those participants in cohort A with no risk factors enriched the predictability of age and BMI, which is consistent with advancing age and higher BMI as risk factors for developing heart failure. These data suggest that PNC has diagnostic value for subclinical heart failure.

### PNC Is Positively Correlated to NT‐proBNP

**Figure 3:**
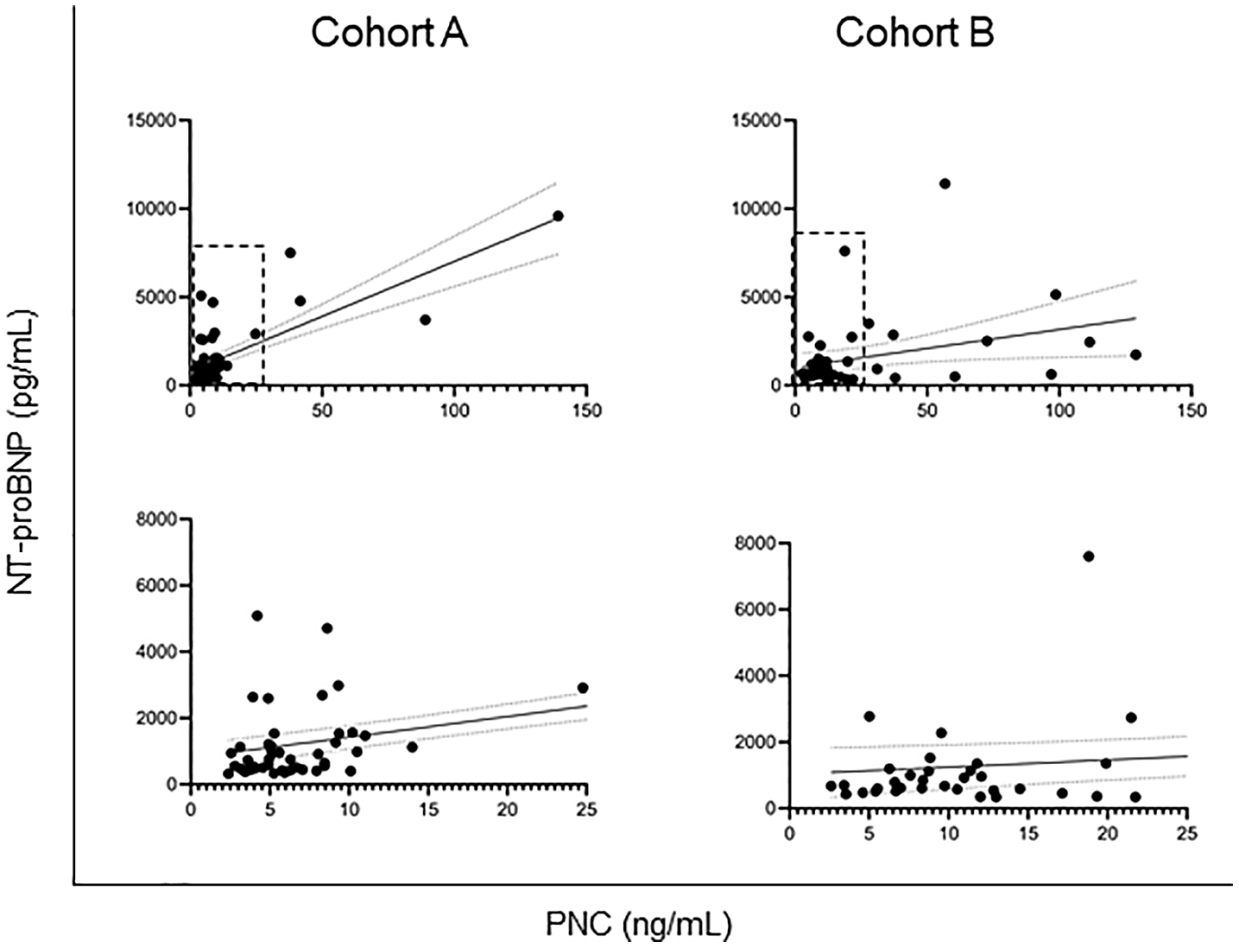
PNC values correlate to NT‐proBNP values. (Top) Simple linear regression was used to determine a correlation between PNC levels and NT‐proBNP levels in each cohort. A modest correlation is observed in cohort A (n=51; slope, 62.16; *r* ^2^=0.56; slope, non‐0; *P*<0.0001), and a slight correlation is found in cohort B (n=44; slope, 21.41; *r* ^2^=0.10; slope, non‐0; *P*=0.0326). (Bottom) Inset of hashed area of top graphs. NT‐proBNP indicates N‐terminal pro‐B‐type natriuretic peptide; and PNC, precursor pro‐N‐cadherin.

The relationship between PNC and NT‐proBNP in these cohorts was investigated. Participants' PNC and NT‐proBNP levels from each cohort were analyzed by simple linear regression using data of participants whose NT‐proBNP values were within the range of the assay. Interestingly, a positive correlation between PNC and NT‐proBNP is found within cohort A (Figure 3, left panels; slope 62.16; *r* ^2^=0.56); however, a weaker correlation is observed in cohort B (Figure 3, right panels; slope 21.41; *r* ^2^=0.10). These data suggest a correlation between PNC and NT‐proBNP serum levels.

### PNC Levels Are Correlated With All‐Cause Mortality

**Figure 4:**
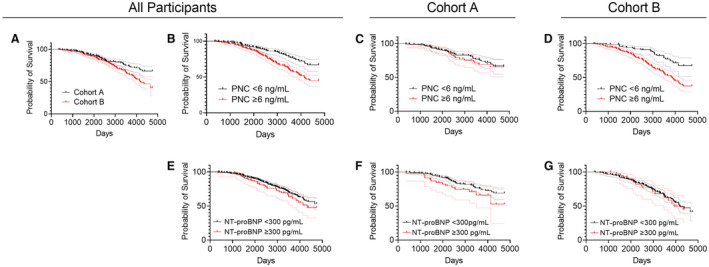
Prognostic value of PNC levels demonstrated by survival curves. Comparison of survival curves was analyzed using the log‐rank test. **A**, Cohort B has a significantly lower 13‐year survival rate than cohort A (total n=596; log‐rank HR, 1.64 [95% CI, 1.22–2.20]; *P*=0.0016). **B**, Participants from combined cohorts measuring PNC levels ≥6 ng/mL have significantly lower 13‐year survival than participants measuring PNC levels <6 ng/mL (total n=596; log‐rank HR, 1.99 [95% CI, 1.48–2.67]; *P*<0.0001). **C**, There is no significant difference between survival curves of cohort A between participants whose PNC levels measure ≥6 ng/mL and participants measuring <6 ng/mL (total n=289; log‐rank HR, 1.17 [95% CI, 0.69–1.98]; *P*=0.5465). **D**, Participants from cohort B measuring PNC levels ≥6 ng/mL have significantly lower 13‐year survival than participants measuring PNC levels <6 ng/mL (total n=307; log‐rank HR, 2.53 [95% CI, 1.74–3.69]; *P*<0.0001). **E**, No significant difference was found in 13‐year survival between participants from combined cohorts measuring NT‐proBNP levels ≥300 pg/mL relative to participants measuring NT‐proBNP levels <300 pg/mL (total n=590; log‐rank HR, 1.27 [95% CI, 0.84–1.92]; *P*=0.2098). **F**, No significant difference was found in 13‐year survival between participants from cohort A measuring NT‐proBNP levels ≥300 pg/mL relative to participants measuring NT‐proBNP levels <300 pg/mL (total n=289; log‐rank HR, 1.66 [95% CI, 0.82–3.33]; *P*=0.0931). **G**, No significant difference was found in 13‐year survival between participants from cohort B measuring NT‐proBNP levels ≥300 pg/mL relative to participants measuring NT‐proBNP levels <300 pg/mL (total n=301; log‐rank HR, 1.05 [95% CI, 0.63–1.75]; *P*=0.8507). Each curve is depicted as the probability of survival and the 95% CI. HR indicates hazard ratio; NT‐proBNP, N‐terminal pro‐B‐type natriuretic peptide; and PNC, precursor pro‐N‐cadherin.

Survival curves were constructed using measured PNC or NT‐proBNP levels and the days after sample collection to the reported death dates or days after sample collection to the last follow‐up year recorded over a total of 13 years. Initially, we compared the overall survival of cohort A to cohort B, and, as predicted, there was a significant reduction in the survival rate of cohort B relative to cohort A (Figure 4A; HR, 1.64 [95% CI, 1.22–2.20]; *P*=0.0016). Then we assigned a PNC level threshold value of 6 ng/mL, which falls between the median values for PNC levels of both cohorts. There is a significant reduction in the 13‐year survival rate for participants from combined cohorts A and B whose PNC level measures ≥6 ng/mL (Figure 4B; HR, 1.99 [95% CI, 1.48–2.67]; *P*<0.0001). There is no significant difference between 13‐year survival of participants within cohort A who measured >6 ng/mL versus participants who measured <6 ng/mL for PNC (Figure 4C). However, there is a significant reduction in 13‐year survival in participants that measure >6 ng/mL in cohort B relative to those who measure <6 ng/mL in cohort B (Figure 4D; HR, 2.53 [95% CI, 1.74–3.69]; *P*<0.0001). As a combined cohort, we found no significant difference in survival between individuals that measured <300 pg/mL NT‐proBNP and those who measured >300 pg/mL (Figure 4E). Although not significant, there is a reduction in survival for participants whose NT‐proBNP measures >300 pg/mL in cohort A (Figure 4F; HR, 1.66 [95% CI, 0.82–3.33]; *P*=0.093). There is no significant difference in survival for participants whose NT‐proBNP measures <300 pg/mL versus >300 pg/mL in cohort B (Figure 4G). Additional analysis was performed using the Cox proportional hazards ratio. Age, BMI, and PNC are significant variables relative to all‐cause mortality and development of heart failure after adjustment for all other risk covariates (Table 3). These data suggest that PNC has diagnostic/prognostic value for subclinical heart failure within the general population.

**Table 3:** Cox Proportional Hazards Regression Analysis of Combined Cohorts (n=596)

Variable	Hazard ratio	95% CI	*P* value
All‐cause mortality model			
Age, y	1.098	1.078 to 1.119	<0.0001
BMI	1.037	1.009 to 1.064	0.0077
Sex, female	0.9452	0.6829 to 1.312	0.7348
PNC [≥6 ng/mL]	1.414	1.014 to 1.993	0.044
NT‐proBNP [≥300 pg/mL]	1.22	0.8032 to 1.794	0.33
High blood pressure	1.231	0.8592 to 1.796	0.2676
Heart attack	0.8792	0.5097 to 1.484	0.6362
Coronary artery disease	1.276	0.7762 to 2.032	0.3205
Heart failure model			
Age, y	1.008	0.9963 to 1.021	0.1764
BMI	1.020	1.002 to 1.037	0.0232
Sex, female	1.228	0.9586 to 1.578	0.1060
PNC [≥6 ng/mL]	1.555	1.213 to 2.002	0.0006
NT‐proBNP [≥300 pg/mL]	0.9395	0.6623 to 1.300	0.7161
High blood pressure	1.259	0.9639 to 1.655	0.0950
Heart attack	1.663	1.092 to 2.516	0.0169
Coronary artery disease	1.353	0.9043 to 1.988	0.1326

Hazard ratios are adjusted for all other predictor variables and representative of all‐cause mortality (top) or development of heart failure (bottom). BMI indicates body mass index; NT‐proBNP, N‐terminal pro‐B‐type natriuretic peptide; and PNC, precursor pro‐N‐cadherin.

## Discussion

Studies consistently report the prognostic value of NT‐proBNP for patients with heart failure; by contrast, studies showing the prognostic value of NT‐proBNP in individuals with subclinical heart failure in the general population are inconsistent. NT‐proBNP is a poor diagnostic tool for screening of subclinical heart failure in the general population in 2 recent studies of large cohorts.^23^, ^24^ NT‐proBNP was evaluated as a means to predict those with stage B heart failure defined by 12‐lead ECG and Doppler transthoracic echocardiogram from a healthy population and was found ineffective (AUC, 0.566).^23^, ^24^ In addition, NT‐proBNP was found to have no prognostic value in predicting overall survival in a long‐term follow‐up study with a large cohort of healthy participants.^16^ In part, this lack of prognostic value can be attributed to a common single nucleotide polymorphism found within the promotor region of BNP that results in elevated BNP products in the blood.^25^ However, this does not fully explain the complexity of NT‐pro/BNP as a biomarker.

Another challenge for clinicians when considering NT‐pro/BNP as part of heart failure diagnosis is the lack of standardization. While 100 pg/mL is a widely agreed‐upon rule‐out concentration for BNP, there is otherwise considerable variability and a large gray zone.^11^, ^26^ This is further complicated by lack of standardization between assays currently in use for clinical applications.^11^, ^26^ Despite this, in one of the most cited studies describing NT‐proBNP, the age‐dependent rule‐in consensus values of 450, 900, and 1800 pg/mL for ages <50, 50 to 75, and >75, respectively, yielded 90% sensitivity and 84% specificity for acute heart failure.^22^ The consensus <300 pg/mL had a negative predictive value of 98% in the same study.^22^ While this is helpful, there is a clear unmet need for a biomarker to identify patients at risk for developing heart failure before onset of symptoms.

Finally, NT‐pro/BNP must also be evaluated through the lens of other comorbidities and physiological variables that are known to raise or lower peptide concentrations. Standard NT‐pro/BNP levels are significantly different between races and dependent on BMI.^8^, ^12^, ^27^, ^28^ Advancing age, female sex, renal dysfunction, atrial fibrillation, and inflammation are characteristics contributing to high serum NT‐proBNP, while obesity leads to low serum concentration, which can make interpretation difficult.^7^, ^10^, ^13^, ^29^ Approximately 50% of heart failure cases are classified as heart failure with preserved ejection fraction, in which a majority of patients maintain normal natriuretic peptide levels.^30^, ^31^, ^32^ Taken together, these factors are particularly problematic when considering medically at‐risk populations who more often face challenges being correctly diagnosed and having access to appropriate care. Including other biomarkers, such as PNC, provides the opportunity to improve the efficiency and accuracy of care, particularly when confounding variables are present. Future studies are needed to elucidate relationships between PNC and confounding variables not explored in this study and to determine the usefulness of PNC as a potential biomarker for heart failure with preserved ejection fraction.

BNP and NT‐proBNP have proven to be sufficient biomarkers for ruling in and ruling out heart failure in patients already presenting with dyspnea; however, there is a clear need for biomarkers that predict heart failure earlier in disease progression to allow for intervention before remodeling becomes irreversible. This is evidenced by the increased mortality and incidence of sudden death associated with subclinical heart dysfunction.^33^, ^34^ In our study, NT‐proBNP levels >300 pg/mL were not prognostic for survival (Figure 4). In part, this may be attributable to the relatively low incidence of heart failure risk factors at baseline in these participants (Table 1, cardiovascular risk factors). These data suggest that PNC is elevated in the serum during early cardiac remodeling with predictive value for heart failure independent of existing comorbidities. Therefore, it could be used to identify patients who would benefit from preventative or early interventional therapy. Of note, ≥6 ng/mL PNC in the serum was not predictive of 13‐year survival in cohort A, and those individuals in cohort A with PNC >6 ng/mL have not been evaluated for other pathologies or unreported cardiomyopathy in the context of this study. Given that our previous work showed that fibrosis in other organs such as the lungs and liver result in serum PNC in the range of 4 to 6 ng/mL,^17^ it is possible that serum PNC levels in these individuals may be attributable, at least in part, to other pathologies. This also suggests that a certain threshold of PNC in the serum may be necessary to become predictive of heart failure and diverge from that of other pathologies.

Our data specifically indicate that PNC ≥6 ng/mL increases the probability of developing heart failure and all‐cause mortality in the cohort of individuals analyzed. As is the case with any clinical biomarker, it will be important to understand what range of normal values exists within the general population and what other pathologies, polymorphisms, or syndromes may contribute to the development of PNC levels that fall outside of the “normal” range. Further work is needed to determine the utility of serum PNC prognostication in the context of other pathologies or high PNC that is otherwise not attributable to heart failure.

Serum PNC is indicative of the aberrant processing, localization, and solubilization of PNC from the cell surface observed in pathological tissue remodeling and fibrosis.^17^ Our findings indicate that PNC is detectable in serum before the stage at which tissue fibrosis and remodeling produces increased cardiac wall tension and elevated NT‐pro/BNP. This suggests that PNC could be used as a predictive screening biomarker for subclinical heart failure of at‐risk individuals within the general population. Our data indicate that a community‐based screening approach of individuals measuring >8.13 ng/mL PNC results in a sensitivity of 77.8% and specificity of 77.5% that these individuals will be diagnosed with heart failure within 2 years. Additionally, we found that individuals with serum PNC levels ≥6 ng/mL have a 41% increased chance of all‐cause mortality after adjusting for age, sex, BMI, NT‐proBNP level, presence of high blood pressure, heart attack, and coronary artery disease. Taken together, these data suggest that PNC is a practical screening tool to identify individuals with cardiovascular risk factors commonly found in the general population and older individuals that will progress to heart failure. There is no established biomarker known to the literature that is predictive of heart failure independent of age, sex, BMI, or comorbidities that can be used clinically as a community‐based screening tool for subclinical heart failure.^24^, ^35^ Because this is the first report with evidence of serum PNC as a biomarker for subclinical heart failure, more studies will be necessary to establish clinical cutoffs and practical utility of serum PNC as a biomarker of subclinical heart failure in the clinical setting.

It is important to note that this study was limited by the nature of a self‐reporting study. Limited information pertaining to clinical factors and comorbidities was available. While our data indicate that PNC adds predictive value to the study‐designated cardiovascular risk factors, survival was analyzed on the basis of all‐cause mortality and could not be definitively attributed to cardiovascular‐related death. No echocardiogram data were available to exclude participants with cardiac structural anomalies or asymptomatic heart disease from cohort A. Furthermore, with only 1 blood collection (at time of enrollment in the study), the dynamics of serum PNC over time could not be evaluated over the decade or more of participant follow‐ups. Nonetheless, the significance of the ability to detect elevated PNC in a population with no self‐reported heart failure before the onset of diagnostic symptoms should not be understated. Future studies are warranted to establish the prognostic potential of soluble PNC in a prospective manner.

## Sources of Funding

The MURDOCK study was funded by a gift from the David H. Murdock Institute for Business and Culture and is supported by Duke's National Institutes of Health National Center for Advancing Translational Sciences Clinical and Translational Science Award UL1TR002553. Authors P. Ferrell, I. Puranam, and Dr Pizzo received support from the Duke University Kaganov Research Initiative in Pulmonary Medicine and Engineering, FY20; and authors P. Ferrell and Dr Pizzo received support from the North Carolina Biotechnology Center, 2019‐TEG‐1505.

## Disclosures

Authors P. Ferrell and Drs Oristian and Pizzo are inventors on unlicensed US patent(s) held by Duke University and related to work discussed in this article.

## Supporting information

Data S1
Figure S1
