# Supplementary material for: Serum Pro‐N‐Cadherin Is a Marker of Subclinical Heart Failure in the General Population
Source: J Am Heart Assoc. 2023 Mar 9;12(6):e028234. doi: 10.1161/JAHA.122.028234 (PMC10111553; doi:10.1161/JAHA.122.028234)

SUPPLEMENTAL MATERIAL

Data S1. Measurement to Understand Reclassification of Disease Of Cabarrus and Kannapolis (MURDOCK) Study Storefront participants with cardiovascular disease. Reprinted with permission from the MURDOCK storefront (https://ctsi.duke.edu/research-support/duke-kannapolis/murdock-study).

MURDOCK STUDY

Managed by Duke Clinical & Translational Science Institute

The MURDOCK Study Community Registry and Biorepository is a 12,526-participant community-based longitudinal cohort recruited from a 20-Zip Code region in the Southeastern United States (U.S.) that is centered in the city of Kannapolis, NC and encompasses Cabarrus County, NC.

Creation of the cohort was funded by a gift to Duke University from the David H. Murdock Institute for Business and Culture, with operational support from Duke's Clinical and Translational Science Award (CTSA) grant (UL1TR002553) and the Duke Clinical and Translational Science Institute (CTSI).

Consenting participants complete a baseline health questionnaire at enrollment, as well as a brief physical exam and collection of blood and urine. Consent includes permission to access to information from medical records, storage of collected samples in the biorepository, access to collected data and biospecimens for future approved research studies and contact regarding new research study opportunities.

Data have been organized into "storefronts" that summarize characteristics of a population of research interest as well as available data and samples for that population. The following sections summarize the sources of data in the MURDOCK Study database, as well as important descriptions and definitions to help understand the data presented in the "storefronts".

1 Participant self-reported data at baseline. The baseline questionnaire collects contact information, current residential street address, and primary physician; alternate contact information; date and place of birth; demographics; current or past diagnosis of 34 medical conditions; menopausal status in women; medications, vitamins and supplements; dietary and physical activity assessment; hours of sleep per night; tobacco and alcohol use; second-hand smoke exposure; and selected PROMIS® participant-reported outcomes domains. Socioeconomic data collected at baseline included marital status, highest level of education of participant and participant's mother and father, employment status, mother's and father's occupations, housing (type, how paid for, number of adults and children in the household) and total household income. In addition, a brief physical exam (vital signs, height, weight, and waist circumference) was conducted at enrollment.

Medical conditions: "Do you **have, or have you ever had**, any of the following [medical conditions]?" (yes, no, don't know). Counts are unique participants reporting yes to specific condition. **Medications**: "Please list any pharmaceutical and/or natural medications (including vitamins) that you are currently taking." Data are captured in free-text format as written by the participant and coded using RxNorm. Summary metrics are based on everything reported. Top 5 reported medications are limited to reported prescriptions.

2 Biorepository samples. Blood was collected at baseline and processed into the following specific samples: whole blood in EDTA for DNA extraction, whole blood in PAXgene for RNA extraction, plasma, serum and buffy coat in cryovials. Urine was collected and aliquoted in cryovials. Sample collection was not done systematically for MURDOCK enrollees; however, some nested sub cohorts and other studies enrolling MURDOCK registry participants include sample collection at follow up time points. All samples are stored at -80°C in a central biorepository current managed by Fisher BioServices, a division of Thermo Fisher Scientific, under a contractual agreement with Duke University.

Samples in inventory: Data are summarized by sample type as well as specific container and size. Participant counts are unique individuals with one ore more aliquots. Aliquot counts are all unique samples for a given type and container, size. Freezers is a calculation of approximate storage requirements based on sample type/size, box size, and number of boxes that can be stored per freezer.

3 Participant self-reported changes in health via annual follow up. Participants are asked to complete a follow-up form once a year around the time of their original enrollment date. Participants may update contact information, primary care physician/practice and alternate contact. PROMIS domains are repeated at each annual time point in order to capture changes in participant-reported outcomes over time. The form collects new incidence/diagnosis of the same 34 medical conditions surveyed at baseline. Hospitalizations during the past year are collected along with reason, as well as specific medical procedures. Participants may update their medication list to reflect current medications, vitamins and supplements being taken at the time of follow up form completion.

Vital status: Death reported by family member or alternate contact is confirmed by obituary as the primary source. Cause of death is not captured. **Follow-up metrics**: Follow-up is defined as complete if participant fills out the survey online or by mail or phone. Completeness is measured as surveys completed relative to years eligible to complete follow-up. **Medical conditions**: "Please indicate if you have received a **new** diagnosis of any of the following medical conditions **in the past year** (yes, no, don't know)". Counts and percentages are unique participants reporting yes to specific condition in follow-up for participants that did NOT report yes at baseline. **Procedures**: "Please indicate if you have any of the following medical procedures **in the past year**". Counts are unique participants reporting the specified procedure one or more times during follow up. **Hospitalizations**: Participants are asked to report if they have been hospitalized **within the last year**, for each hospitalization they are asked to list reason(s) for hospitalization, admission date and hospital name. Reasons for hospitalization are captured as free-text responses as written by participants. Responses are coded, when possible, in order to list the most frequently reported reasons for hospitalization. **Medications**: (see note above for medications reported at baseline). The denominator for data based on last follow-up are participants with at least one follow-up survey complete.

4 Electronic health record (EHR) data from regional healthcare providers. Duke has partnered with regional healthcare providers to integrate data from EHR systems for consented MURDOCK Study participants. Participants are identified in EHR systems with robust matching algorithms using common identifiers from the MURDOCK and EHR databases. Data are transferred under a data use agreement (DUA) with the specific provider organization which specifies the scope of data and frequency of transfers. Data availability vary by participant and depend on whether or not a participant has had one or more encounters with the healthcare provider system during the time period included in the dataset.

Available EHR datasets: Data are summarized by healthcare provider organizations. Counts are unique participants with one or more ICD codes in the EHR dataset. **Available EHR domains**: Data area summarized by domain in the EHR dataset. Counts are unique participants with one of more records (rows of data) for the specified domain. **Insights from available EHR data**: Specific EHR data related to the population of research interest is presented with granularity when possible.

5 Additional data collection from studies with MURDOCK participants. MURDOCK Study participants may be recruited to enroll in additional research study opportunities by Duke researchers or other collaborators. Data sharing is a condition of collaboration with with the MURDOCK Study; therefore, data collected from MURDOCK Study participants and/or generated from biospecimens as part of additional research studies is returned for integration with all other MURDOCK registry data.

"Storefronts" for nested sub-cohorts summarize surveys, assessments and/or other data collected specifically as part of enrollment and participation in the study. **Samples in inventory**: Samples are summarized if collected (see note above for samples collected at baseline). **Participation in other studies**: Counts are participants from the population of research interest enrolled in the specified study listed. *Brief descriptions of relevant studies are listed along with a summary of study procedures and/or data collected.*

MURDOCK Study participants with cardiovascular disease, N=2,798

Participant self-reported characteristics at MURDOCK Study enrollment (baseline, [February 2009 - February 2018])

**CVD Phenotypes in the MURDOCK Study**	
Atrial fibrillation	1,059
Heart failure	650
Peripheral arterial disease	84
Stroke	721

**Demographics at baseline**	
**Age**	**Baseline**
Median (25th, 75th)	65 (56, 73)
Min, Max	<18, 90+
**Sex**	
Female	1,494 (53%)
Male	1,304 (47%)
**Race**	
American Indian & Alaska Native	10 (<1%)
Asian	4 (<1%
Black or African American	325 (12%)
Native Hawaiian & Other Pacific Islander	1 (<1%)
White/Caucasian	2,321 (83%)
Other	67 (2%)
Multiple	56 (2%)
Don't know/Not sure/Not answered	14 (1%)
**Ethnicity**	
Hispanic or Latino	110 (4%)
Non-Hispanic or Latino	2,641 (94%)
Don't know/Not sure/Not answered	47 (2%)

**Smoking history at baseline**	
Smoked	1,520 (54%)
Never smoked	1,255 (45%)
Don't know, no response	23 (1%)

Current or prior medical conditions reported at baseline
20 of 34 solicited medical conditions, listed by descending frequency

High blood pressure	1,719
High cholesterol	1,683
Obesity	878
Osteoarthritis	817
Depression	777
Diabetes	752
Coronary artery disease	712
Heart attack or angina	689
Skin cancer, not melanoma	549
Atrial fibrillation	542
Thyroid disease	463
Osteoporosis/Osteopenia	438
Asthma	420
Stroke	360
Rheumatoid arthritis	325
Congestive heart failure	291
Emphysema or "COPD"	288
Gout	274
Other autoimmune disease	168 (6%)
Implantable cardiac defibrillator	152 (5%)

**Education at baseline**	
Less than high school graduate	256 (9%)
High school graduate, equivalent	694 (25%)
Some college or associates degree	1,060 (38%)
Bachelor's degree	476 (17%)
Master's or higher professional degree	308 (11%)

**Income at baseline**	
Under $10,000	192 (7%)
$10,000-29,999	630 (23%)
$30,000-49,999	530 (19%)
$50,000-69,999	436 (16%)
$70,000-89,999	277 (10%)
$90,000 or more	433 (15%)
Don't know, no response	300 (10%)

**Body mass index (BMI) at baseline**	
<18.5 (underweight)	32 (1%)
18.5 - 24.9 (normal weight)	641 (23%)
25 - 29.9 (overweight)	1,013 (36%)
30+ (obese)	1,103 (40%)

**Exercise at baseline**	
Little to no physical activity	1,293 (46%)
Weekend light exercise	375 (13%)
Moderate activity 3x per week	756 (27%)
Heavy activity 3x per week	203 (7%)
Heavy activity 5x per week	149 (5%)

**Medications, vitamins, supplements at baseline**	
Median (25th, 75th) reported	9 (5, 12)
10+ reported, n (%)	1,192 (43%
**Top 5 reported medications (coded)**	
Lisinopril	647 (23%
metoprolol	526(19%
simvastatin	523 (19%
omeprazole	511 (18%
hydrochlorothiazide	442 (16%

Samples in inventory, collected at baseline

Sample	Container, Size	Participants	Aliquots	Freezers
Plasma	Cryovial, 0.5 mL	2,608	33,327	0.587
	Cryovial, 4.0 mL	0	0	0
Serum	Cryovial, 0.5 mL	2,613	21,676	0.382
	Cryovial, 4.0 mL	0	0	0
	Cryovial, 5.0 mL	2,320	2,321	0.081
Whole blood	PAXgene RNA	2,450	5,212	0.303
	Vacutainer, 2.0 mL	1,144	1,737	0.050
	Vacutainer, 3.0 mL	0	0	0
	Vacutainer, 4.0 mL	0	0	0
Buffy coat	Cryovial, 2.0 mL	1,633	1,634	0.028
Urine	Cryovial, 0.5 mL	7	7	0.0001
	Cryovial, 4.0 mL	0	0	0
	Cryovial, 10.0 mL	2,478	7,692	0.610
Total				2.0411

MURDOCK STUDY

Managed by Duke Clinical & Translational Science Institute

MURDOCK Study participants with cardiovascular disease, N=2,798

Participant status and data from MURDOCK Study follow-up surveys and electronic health records

**Participant vital status**	
Alive	2,125 (76%)
Deceased	673 (24%)

**Current Age**	**Current**
Median (25th, 75th)	73 (64, 80)
Min, Max	25, 90+

**Follow-up metrics, study participation**	
Median (25th, 75th) months since enrollment	129 (110, 144)
Median (25th, 75th) years since enrollment	11 (9, 12)
Median (25th, 75th) annual follow-ups complete	6 (3, 9)
Overall completeness of follow-up, n/N (%)	15,771/22,322 (71%)
At least one (1) follow-up survey complete, n (%)	2,530 (90%)
100% completion (n, %)	1,040 (37%)
Last completed follow-up ≤ 18 months	1,360 (49%)
Enrolled in one or more other studies	1,449 (52%)

**Available EHR datasets by source (any ICD code)**	
Any source	1,288 (46%)
Novant Health	944 (34%)
Cabarrus Health Alliance	403 (14%)
Cabarrus Rowan Community Health Centers	90 (3%)
Bethesda Health Center	13 (<1%)
Community Free Clinic	11(<1%)
Atrium (Carolinas Healthcare)	0

**Available EHR data domains**	
Diagnoses	1,288 (46%)
Labs	1,003 (36%)
Vitals	915 (33%)
Medications	970 (35%)
Allergies	607 (22%)
Immunizations	487 (17%)
Problems	809 (29%)
Procedures	597 (21%)
Hospitalizations	478 (17%)

Insights from available EHR data

Date range: July 1993 (first encounter), Jan. 2021 (last encounter)

Number of days between first and last encounter:

Median (25th, 75th)	1757 (223, 2,881)
Min, Max	0, 10,034

Phecode	*Description*	*Group*	*n, ppts*
401.1	Essential hypertension	circulatory system	440
272.1	Hyperlipidemia	endocrine/metabolic	439
250.2	Type 2 diabetes	endocrine/metabolic	197
411.4	Coronary atherosclerosis	circulatory system	174
530.1	Esophagitis, GERD	endocrine/metabolic	144
261.4	Vitamin D deficiency	endocrine/metabolic	139

Select laboratory tests

Test	*Labs*	*Participants*
Comprehensive metabolic panel	4,681	626
CBC and differential	3,427	569
Basic Metabolic Panel	3,547	539
Lipid Panel	2,442	528
TSH	2,167	497
Hemoglobin A1c	2,601	495

New medical condition diagnoses reported in follow-up
17 of 34 solicited medical conditions, listed by descending frequency

Atrial fibrillation	485 / 2,256 (21%)
Osteoarthritis	449 / 1,981 (23%)
Coronary artery disease	421 / 2,086 (20%)
High cholesterol	341 / 1,115 (31%)
Rheumatoid arthritis	315 / 2,473 (13%)
Skin cancer, not melanoma	302 / 2,249 (13%)
Stroke	301 / 2,438 (12%)
Congestive heart failure	294 / 2,507 (12%)
Heart attack or angina	286 / 2,109 (14%)
Osteoporosis/Osteopenia	284 / 2,360 (12%)
High blood pressure	277 / 1,079 (26%)
Emphysema or "COPD"	244 / 2,510 (10%)
Depression	234 / 2,021 (12%)
Thyroid disease	225 / 2,335 (10%)
Diabetes	225 / 2,046 (11%)
Obesity	216 / 1,920 (11%)
Kidney disease	199 / 2,672 (7%)

**Procedures reported in follow up**	
CT or MRI scan	2,012 (72%)
Chest x-ray	1,838 (66%)
Joint x-ray	1,551 (55%)
Heart/cardiac stress test	1,351 (48%)
Heart/cardiac catheterization	677 (24%)
Joint replacement	475 (17%)
Heart/cardiac angioplasty or stent	414 (15%)
Coronary artery bypass surgery	187 (7%)

**Hospitalizations reported in follow up**	
Participants reporting 1 or more hospitalizations	1,632 (58%)
Unique hospitalizations reported	2,919
Median (25th, 75th) hospitalizations reported	2 (1, 3)

Coded reasons for self-reported hospitalization *listed in descending frequency*	Events	Participants
Uncoded	2,000	1,024
Surgery	351	264
Knee Replacement	218	164
Stroke	211	172
AFIB	209	153

**Body mass index (BMI) at most recent completed follow up**	
<18.5 (underweight)	44 (2%)
18.5 - 24.9 (normal weight)	666 (26%)
25 - 29.9 (overweight)	916 (36%)
30+	896 (36%)

**Medications, vitamins, supplements at most recent follow up**	
Median (25th, 75th) reported	8 (5, 12)
10+ reported, n (%)	945 (34%)

**Top 5 reported medications**	
Metoprolol	620 (22%)
Atorvastatin	616 (22%)
Lisinopril	456 (16%)
Omeprazole	433 (15%)
Levothyroxine	432 (15%)

MURDOCK Study participants with cardiovascular disease, N=2,798

Cardiovascular disease phenotypes in the MURDOCK Study

Atrial fibrillation	n=1,059
**Source of diagnosis**	
Self-report only	939
Self-report & EHR	90
EHR only	30

Samples in inventory, collected at baseline

Sample	Container, Size	Participants	Aliquots	Freezers
Plasma	Cryovial, 0.5 mL	998	12,623	0.222
	Cryovial, 4.0 mL	0	0	0
Serum	Cryovial, 0.5 mL	992	8,063	0.142
	Cryovial, 4.0 mL	0	0	0
	Cryovial, 5.0 mL	885	885	0.031
Whole blood	PAXgene RNA	937	1,916	0.111
	Vacutainer, 2.0 mL	388	584	0.017
	Vacutainer, 3.0 mL	0	0	0
	Vacutainer, 4.0 mL	0	0	0
Buffy coat	Cryovial, 2.0 mL	579	579	0.010
Urine	Cryovial, 0.5 mL	4	4	0.000
	Cryovial, 10.0 mL	943	2,833	0.224
Total				0.757

Heart failure	N=650
**Source of diagnosis**	
Self-report only	548
Self-report & EHR	32
EHR only	26

Samples in inventory, collected at baseline

Sample	Container, Size	Participants	Aliquots	Freezers
Plasma	Cryovial, 0.5 mL	571	7,789	0.137
	Cryovial, 4.0 mL	0	0	0
Serum	Cryovial, 0.5 mL	568	4,786	0.084
	Cryovial, 4.0 mL	0	0	0
	Cryovial, 5.0 mL	486	511	0.018
Whole blood	PAXgene RNA	532	1,216	0.070
	Vacutainer, 2.0 mL	245	404	0.011
	Vacutainer, 3.0 mL	0	0	0
	Vacutainer, 4.0 mL	0	0	0
Buffy coat	Cryovial, 2.0 mL	356	384	0.006
Urine	Cryovial, 4.0 mL	0	0	0
	Cryovial, 10.0 mL	532	1,763	0.139
Total				0.465

Stroke	n=721
**Source of diagnosis**	
Self-report only	632
Self-report & EHR	25
EHR only	31

Samples in inventory, collected at baseline

Sample	Container, Size	Participants	Aliquots	Freezers
Plasma	Cryovial, 0.5 mL	640	8,398	0.148
	Cryovial, 4.0 mL	0	0	0
Serum	Cryovial, 0.5 mL	640	5,543	0.097
	Cryovial, 4.0 mL	0	0	0
	Cryovial, 5.0 mL	567	595	0.020
Whole blood	PAXgene RNA	599	1,346	0.078
	Vacutainer, 2.0 mL	291	452	0.013
	Vacutainer, 3.0 mL	0	0	0
	Vacutainer, 4.0 mL	0	0	0
Buffy coat	Cryovial, 2.0 mL	409	426	0.007
Urine	Cryovial, 0.5 mL	1	1	0.000
	Cryovial, 10.0 mL	605	1,961	0.155
Total				0.518

Peripheral arterial disease	n=84
**Source of diagnosis**	
Self-report only	14
Self-report & EHR	1
EHR only	68

Samples in inventory, collected at baseline

Sample	Container, Size	Participants	Aliquots	Freezers
Plasma	Cryovial, 0.5 mL	79	974	0.017
	Cryovial, 4.0 mL	0	0	0
Serum	Cryovial, 0.5 mL	80	652	0.011
	Cryovial, 4.0 mL	0	0	0
	Cryovial, 5.0 mL	62	63	0.002
Whole blood	PAXgene RNA	75	159	0.009
	Vacutainer, 2.0 mL	33	53	0.001
	Vacutainer, 3.0 mL	0	0	0
	Vacutainer, 4.0 mL	0	0	0
Buffy coat	Cryovial, 2.0 mL	46	46	0.0008
Urine	Cryovial, 4.0 mL	0	0	0
	Cryovial, 10.0 mL	75	245	0.019
Total				0.0598

Figure S1. Graphical representation of the percent distribution of NTproBNP rule-in, rule-out, and "gray zone" of 1-5yr follow-up (left) and 1-2yr follow-up (right) subgroups.

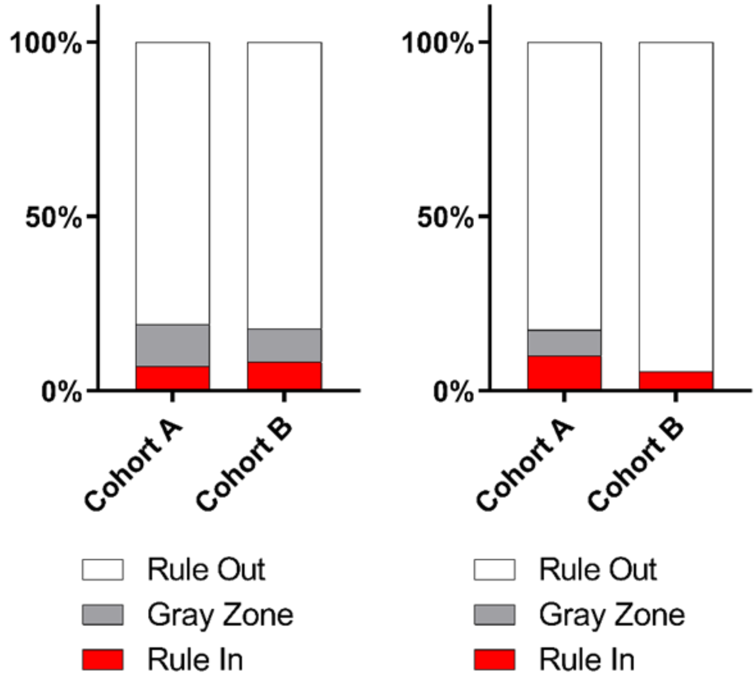

Supplement: Supplementary file 1 — Data S1 Figure S1 [file JAH3-12-e028234-s001.pdf]
